# Intralesional Corticosteroid Injections in the Treatment of Oral Lichen Planus—A Narrative Review

**DOI:** 10.3390/jcm15020561

**Published:** 2026-01-09

**Authors:** Weronika Miazga-Rychlik, Emilia Milczarek, Jan Kowalski, Aniela Brodzikowska, Bartłomiej Górski

**Affiliations:** 1 Department of Periodontology and Oral Mucosa Diseases, Medical University of Warsaw, 6 Binieckiego St., 02-097 Warsaw, Poland; 2 Department of Conservative Dentistry, Medical University of Warsaw, 6 Binieckiego St., 02-097 Warsaw, Poland

**Keywords:** oral lichen planus, corticosteroid injections, triamcinolone acetonide

## Abstract

**Background**: Oral lichen planus (OLP) is a chronic inflammatory autoimmune disease. Certain clinical forms of the disease may cause significant discomfort and negatively impact patients’ quality of life. The first-line treatment is topical corticosteroids. The purpose of this narrative review is to evaluate the influence of corticosteroid injections in the treatment of OLP. **Methods**: A search of the literature was conducted in October 2025 using the following databases: PubMed, Web of Science, Scopus, and Cochrane. **Results**: Fifteen studies were evaluated. Injections with triamcinolone acetonide at a concentration of 10–40 mg/mL at weekly intervals demonstrated efficacy in reducing pain and healing OLP lesions in these studies. They showed minor adverse effects. **Conclusions**: Noting the limitations of this narrative review, intralesional corticosteroid injections are an effective and safe method of treating OLP. They should be considered in patients with symptomatic OLP.

## 1. Introduction

**Figure 1:**
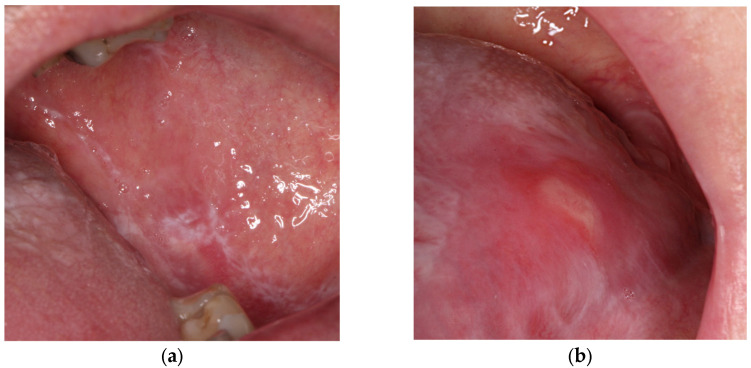
Lesions on oral mucosa in patients with oral lichen planus: (**a**) reticular type; (**b**) erosive type.

Oral lichen planus (OLP) is a chronic inflammatory autoimmune disease with a global prevalence of 1.01%, reaching 1.32% in Europe [1]. OLP is 3–4 times more common in women and the typical age of onset is between 30 and 70 [2]. The etiology of OLP is unknown, but various factors have been associated with this disease: anxiety, diabetes, autoimmune diseases, chronic liver disease, intestinal diseases, increased cholesterol blood levels, medications, stress, hypertension, infections, contact with dental materials, cancer, and a genetic predisposition to cancer [2]. The most common oral locations are the buccal mucosa, tongue, and gingiva, followed by the labial mucosa and vermilion of the lower lip. The distribution of OLP is typically bilateral and symmetric [3]. There are six clinical subtypes of OLP that can be seen alone or in combination: reticular, plaquelike, atrophic, erosive/ulcerative, papular, and bullous [4] (Figure 1). Erythematous and erosive lesions may cause significant pain, burning, swelling, irritation, and bleeding [3]. Erosive OLP is recognized as a potentially malignant disorder, associated with a risk of developing oral squamous cell carcinoma (SCC), with an overall malignant transformation rate estimated at approximately 1.4% [5].

Reticular lesions that are asymptomatic generally do not require therapy, but only close observation. Management should focus on treating atrophic and erosive/ulcerative lesions [4]. The first-line treatment of localized OLP is the topical application of steroids: clobetasol propionate, triamcinolone, betamethasone, fluocinonide, fluticasone, dexamethasone, and prednisolone, used in different forms (ointment, oral suspension/aqueous solution, pellets, aerosol/spray, mouthwash, adhesive paste). The intralesional injection of corticosteroids (triamcinolone acetonide, hydrocortisone, dexamethasone, and methylprednisolone) is an effective treatment in ulcerative OLP [6]. Systemic corticosteroids, such as methylprednisolone or prednisone, should be reserved for acute exacerbation, multiple/widespread lesions, and for patients not responding to topical steroids [7]. Other interventions recommended for treatment are systemic (acitretin, isotretinoin) or topical retinoids (isotretinoin or other forms of vitamin A) and oral cyclosporine [6].

Intralesional corticosteroid injections have been explored as a targeted therapeutic approach for symptomatic oral lichen planus, offering potential advantages over systemic or topical treatments, particularly in terms of localized efficacy and reduced systemic exposure. The purpose of this narrative review is to evaluate the influence of corticosteroid injections in the treatment of OLP and to identify the adverse effects of this treatment. Given the heterogeneity of study protocols, small sample sizes, and variable outcome measures in existing research, a narrative review is appropriate to synthesize current evidence and identify gaps in knowledge.

## 2. Materials and Methods

A search of the literature was performed in October 2025 using PubMed, Web of Science, Scopus and Cochrane, with the keyword phrase “corticosteroid injections oral lichen planus”. The literature search specifically focused on studies addressing the treatment of oral lichen planus with intralesional corticosteroid injections. The review was limited to articles published in the last 20 years and focused on primary research articles reporting original clinical data, including randomized controlled trials, cohort studies, case–control studies, and case series. Systematic reviews, meta-analyses, and other narrative reviews were excluded. All selected articles were in English. Extracted data included author and year of publication, study design, study intervention, assessment tools used to evaluate treatment response, reported study outcomes, adverse events, duration of follow-up, and recurrence. Treatment effectiveness was not independently assessed; this review summarizes the outcomes and conclusions reported in the original studies.

## 3. Results

**Figure 2:**
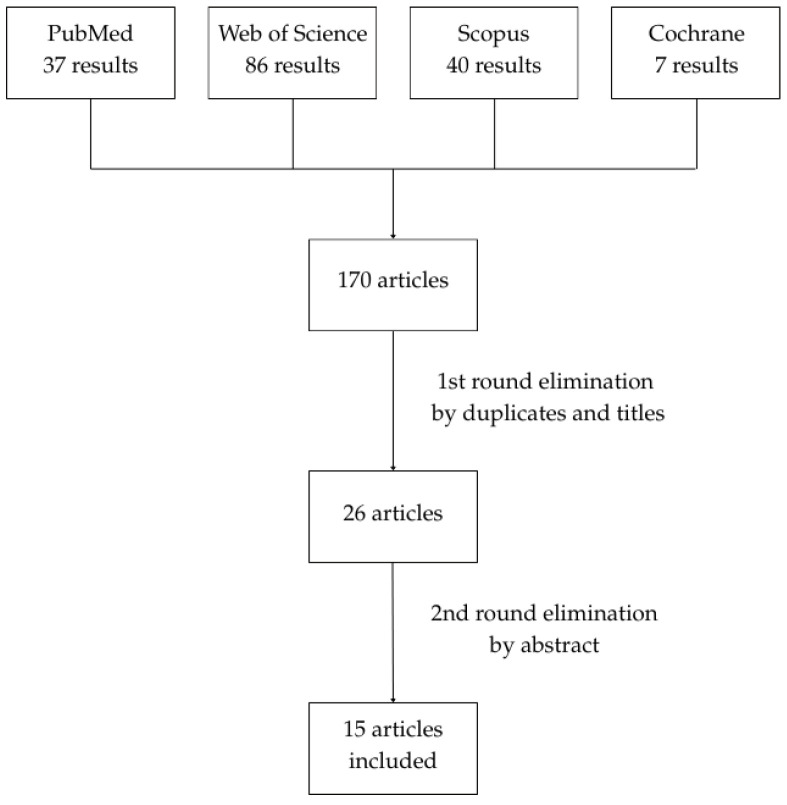
Process of article elimination.

The initial databases screening identified 170 articles. After excluding duplicates, extraoral locations, diseases other than lichen planus, and publications not related to intralesional steroid injections, as well as meta-analyses, systematic reviews, and narrative reviews, 15 articles were selected. The process of article elimination is presented in Figure 2. A list of selected studies is provided in Table 1. The studies in the table are organized by study design and, within each category, roughly chronologically, with related studies placed adjacent to facilitate comparison.

Xia et al. [8] conducted a split-mouth randomized controlled trial on patients with ulcerative OLP, in which an injection of TA with lidocaine was compared with no intervention. If the lesion on the injected side did not regress by ≥81% in size, a second injection was administered. There was a significant reduction in pain in the experimental group and a minimal reduction in the control group. Also, the reduction in the size of the lesion was significant in the experimental group: after 4 weeks, 88.9% of patients showed an >80% reduction in erythematous area and 84.4% reduction in ulceration size.

Two studies compared TA injections with Bacillus Calmette-Guerin polysaccharide nucleic acid extract (BCG-PSN) injections.

Xiong et al. [9] published a randomized controlled trial in which 31 patients with erosive OLP received a BCG-PSN injection every other day for 2 weeks (six injections) and 25 patients received a TA injection every week for 2 weeks (two injections). The study showed no statistical differences in erosive areas and VAS scores, recovery rates, and intervals between the two groups. Adverse effects were limited to swelling and burning sensations in 9.7% of the BCG-PMN group and 8.0% of the TA group.

Metwalli et al. [10] conducted a similar study on 26 patients with erosive–ulcerative and atrophic OLP. This study also showed no statistically significant differences between two groups in the reduction in erosion areas, REU scores, and reduction in pain. There were non-significant differences in adverse effects and recurrence rate. The results of both these studies were consistent.

Lee et al. [11] published a randomized controlled trial comparing triamcinolone acetate (TA) mouthrinse and TA intralesional injections on patients suffering from OLP. Both treatment methods resulted in a similar pain reduction in patients, but the improvement in the first week was significantly higher after the TA injections. Furthermore, the objective score of OLP did not reveal significant differences between groups. Importantly, the mouthwash group was more likely to develop side effects (44%—candidiasis), while in the injection group, only 5% developed Cushingoid features. Relapse time was similar in both groups.

Liu et al. [12] conducted a randomized controlled trial comparing injections of TA and betamethasone on patients with erosive OLP. The healed percentage was higher in betamethasone (93.1%) than in TA (66.7%). Also the final reduction in erosion area was greater in the betamethasone group. Moreover, the recurrence rate for the betamethasone group was significantly lower than in the TA group (14.8% vs. 45%, respectively). Pain reduction was similar in both groups. No serious adverse effects were reported.

Ahuja et al. [13] administered one injection of TA or platelet-rich plasma (PRP) per week for two months in patients with erosive OLP. Patients in both groups showed a statistically significant improvement (reduction in pain, erythema, and lesion size), with no differences between groups. There was a lower recurrence rate in the PRP group.

ElGhareeb et al. [14] administered one injection of TA or PRP every two weeks for two months in patients with various types of OLP. In this study, there was also a significant decrease in symptoms in both groups, with no differences between groups. However, there was a statistically increase in the frequency of side effects in PRP group (especially pain) and recurrence (100% in PRP group after 6 weeks).

Bennardo et al. [15] conducted a split-mouth randomized controlled trial, with one injection of TA on one buccal side and platelet-rich fibrin (PRF) on another, on patients with symptomatic OLP, with injections taking place once a week for a month. This study also showed no statistically significant differences between the TA and PRF sides.

Al-Hallak et al. [16] conducted a similar study on patients with symptomatic OLP. Despite the lack of statistically significant differences between TA and PRF in the reduction in VAS and REU score, TA showed more effectiveness in the management of OLP lesions. Recurrence rate was the same for both forms of treatment.

Agha-Hosseini et al. [17] published a randomized controlled trial with a split-mouth design, in which she compared hyaluronic acid (HA) injections with TA with TA injections alone in patients with OLP. The study demonstrated better resolution of lesions and symptoms for TA with HA. There was no statistically significant difference in pain reduction. The recovery rate after 6 months was significantly lower on the side injected with HA and TA than with TA alone (11.1% vs. 74.1%, respectively).

Zhao et al. [18] conducted a study to assess the synergistic efficacy and safety of plaque control on erosive non-gingival OLP. In the experimental group, in addition to TA injections, periodontal scaling and oral hygiene instruction were performed. After 2 weeks, a higher healing rate was noted in the experimental group (completely healed percentage 95.8% vs. 69.9%, respectively). Also, greater reductions in erosion size and pain level were found in the experimental group. Also, the decrease in PI (plaque index) and CPI (community periodontal index) were greater in the experimental group. There was no significant difference in relapse rate.

Kuo et el. [19] conducted an observational interventional study in which he treated 50 patients with erosive oral lichen planus (EOLP) with TA injections (40 mg once weekly; 3 weeks for major EOLP (lesions ≥ 1 cm in diameter) and 2 weeks for minor EOLP (lesions < 1 cm)) combined with oral administration of prednisolone (25–30 mg for major EOLP and 15–20 mg for minor EOLP). The patients were then treated with topical 0.1% dexamethasone (once or twice daily) and oral administration of vitamin B9. After 3 weeks, 90 percent of patients (both with minor and major EOLP) showed complete response and 10% showed partial response. Unfortunately, all patients with a complete response showed recurrence of erosive or ulcerative lesion after 3–24 months of follow-up.

Lee et al. [20] published a prospective cohort study examining factors affecting the improvement in or recurrence of OLP after intralesional TA injections. After weekly injections for 4–6 weeks, an improvement was observed in 80.6% of patients. Patients with lesions on the lips were observed to be less responsive to treatment. A total of 58% of patients experienced recurrence, but no risk factors were found.

Walia et al. [21] conducted a prospective observational study to evaluate the improvement in symptomatic OLP using a combination of TA injections, TA orabase, and tacrolimus (TAC) paste. After the implementation of the treatment protocol, 79% of patients achieved complete remission and 21% achieved partial remission. There was also a significant reduction in pain and burning sensation. After another 3 months, 41% of patients showed relapse.

Kurt et al. [22] published a case series describing three patients with OLP. The patients had previously taken systemic corticosteroids, but they were ineffective. Two patients had their amalgam fillings replaced before receiving TA injections. Two patients had completely resolved symptoms, and one was lost to follow-up.

## 4. Discussion

There is no curative treatment for OLP; the aim of the therapy is to reduce symptoms and improve quality of life [23]. Corticosteroids are central to the symptomatic management of the disease. Their pharmacological utility lies primarily in their anti-inflammatory and immunosuppressive effects. Glucocorticoids inhibit inflammation and allergic reactions [24]. They act by suppressing white blood cell function, stabilizing membrane lysozyme, inhibiting plasminogen activation, and reducing the synthesis of inflammatory mediators such as prostaglandins and leukotrienes [25].

The most frequently chosen treatment method is the administration of topical steroids (clobetasol propionate, fluocinonide, triamcinolone acetonide) [26,27,28]. These agents differ in terms of potency, typical concentration, and frequency of application [29,30] (Table 2).

Topical steroids show effectiveness in the treatment of symptomatic OLP and show fewer side effects (secondary candidiasis, dry mouth nausea, mucosal atrophy) than systemic steroids [7,31,32]. Systemic absorption is clinically insignificant [32]. The reduced time of adherence is a main disadvantage, as they are rapidly cleared from the lesion by saliva flow and oral movements, resulting in short contact and uneven drug distribution [33,34]. Aqueous solutions can improve contact time and reach all lesions, regardless of size or depth, but they expose both healthy and diseased mucosa [30]. However, some lesions remain refractory to treatment. Intralesional injections of glucocorticosteroids can overcome the limitations of topical treatment. This is an effective method for delivering a higher drug concentration and for avoiding the adverse effects of systemic administration (insomnia, mood alteration, weight gain, fluid retention) [7,35]. The adverse effects of systemic corticosteroids are common, particularly when used daily for a long time [36].

All studies included in the review showed a significant effect of triamcinolone acetonide injections in the treatment of OLP lesions and in reducing pain. TA injections (0.5 mL, 40 mg/mL, once a week for 4 weeks and one injection after another 2 weeks) showed a lower rate of side effects than a TA mouthwash [11]. The synergistic effect of plaque control and corticosteroid injections (TA, 0.5 mL, 20 mg/mL) is interesting and encourages further studies [18]. In the treatment, corticosteroids other than TA administered by injection should also be considered: betamethasone (1.4 mg once a week for two weeks) showed higher efficacy and lower recurrence rate than TA (7 mg once a week for two weeks) [12]. Combined corticosteroid treatment—locally injected triamcinolone (2 mL, 20 mg/mL, once a week for 2–3 weeks) plus oral prednisolone (15–30 mg daily for 2 weeks)—is also an effective treatment option [19].

Other treatment protocols should also be mentioned. It should be noted that injections with TA (40 mg/mL) in combination with HA (7 mg) demonstrated better resolution of lesions and symptoms and significantly lower recurrent rate than TA alone [17]. A combination of TA injections (0.5 mL, 40 mg/mL, once a week for 4 weeks, and one injection after another 4 weeks), TA orabase (0.1%), and TAC paste (0.03%) also showed significant effects [21]. Treatment with BCG-PSN injections (0.5 mL every other day for 2 weeks) showed similar effects to TA injection (20 mg/mL), and showed similar recovery rates [9,10]. PRP/PRF injections are also an interesting treatment option, although the research results are not clear—PRP/PRF seems to show similar effectiveness in treating lesions and reducing pain, but research results regarding recurrence are inconsistent, which may be due to differences in the protocols used by the researchers [13,14,15,16].

The limitations reported for individual studies are summarized in Table 1. Considerable heterogeneity among the studies reduces the ability to draw firm conclusions. The concentration of triamcinolone acetonide used in intralesional injections varied between 10 and 40 mg/mL, and the number of injections ranged from 1 to 8. Not all studies used objective scoring systems (such as REU [8,10,14,16], the scoring system by Escudier [11,20] or Thongprasom [15,17,21]) [37]. Most studies also relied on patient-reported outcomes, such as VAS [8,9,11,13,15,16,17,20,21] or NRS [10,12,14,18], which are inherently subjective. Also, the follow-up durations varied widely, from 1 month to 1 year, limiting the ability to accurately assess lesion recurrence. In addition, formal sample size calculations were not reported in most studies, except for Zhao et al. [18], who performed a full calculation, and Agha-Hosseini et al. [17], who based the calculation on a pilot study. Sample size is a key determinant of the precision and reliability of study outcomes, as insufficiently sized samples may produce inconclusive or potentially misleading results and increase the risk of a type II error (failing to detect a true effect) [38]. Direct comparison between studies is also challenging because some of them focus on symptomatic OLP in general [10,11,14,15,16,17,20,21,22], while others specifically address erosive–ulcerative forms [8,9,12,13,18,19]. Additionally, lesion locations vary across studies, with some including only buccal mucosa [8,13,15,16,17] and others also considering sites such as the lips or tongue [9,10,11,12,14,18,19,20,21,22], contributing to variability and inconclusive results. Due to these differences, direct comparisons and conclusions are not possible.

Studies comparing different treatment types showed that PRP/PRF and BCG-PSN produced similar effects to TA in treating lesions [9,10,13,14,15,16], whereas betamethasone and TA combined with HA appeared more effective [12,17]. However, these findings are based on single studies and, due to the limited number of participants, heterogeneous protocols, and different OLP types, firm conclusions cannot yet be drawn.

All studies included in this narrative review reported beneficial effects of intralesional corticosteroid injections, suggesting they should be considered for patients with symptomatic oral lichen planus. As some studies indicated a synergistic efficacy of plaque control with intralesional triamcinolone acetonide injections, incorporating oral hygiene measures may enhance outcomes [18]. However, determining a standardized treatment protocol is challenging due to the considerable heterogeneity among the studies. Based on the available evidence, concentrations of 10–20 mg/mL with 2–4 weekly injections appear to be commonly used [9,10,16,18,19] and may provide guidance for clinical practice, although further well-designed studies are needed to establish optimal dosing and treatment schedules. Clinicians should carefully monitor patients’ responses to therapy and adjust treatment protocols accordingly.

## 5. Conclusions

In summary, injections of corticosteroids in treatment of OLP appear effective in reducing pain and symptoms. The therapy is generally well-tolerated and the reported adverse effects are minimal. It should be considered in patients with symptomatic OLP, taking individual patient characteristics and clinical judgment into account. However, due to the heterogeneity of the study protocols, small sample sizes, and variable follow-up durations, long-term effectiveness and recurrence rates remain uncertain. More larger, randomized prospective clinical studies should be conduced to confirm this result and define optimal treatment parameters and modifications, particularly regarding relapse prevention, because, due to the chronic nature of the disease, a longer time without recurrence would improve patients’ quality of life.

## Figures and Tables

**Table 1:** Main characteristics of the selected studies and summary of reported outcomes.

Author & Year	Study Design	Patients	Study Intervention	Assessment Tool	Study Outcome	Adverse Events	Follow-Up Duration	Recurrence	Limitations
Xia et al. 2006 [8]	RCT	45	I: TA injections (1 mL, 20 mg/mL) with lidocaine 2% on one side of oral buccal mucosa. C: no intervention on the other side.	VAS; REU; measuring the erosive area (mm^2^).	Significant reduction in pain and erythematous and ulcerative area on the experimental side and nonsignificant on the control side.	No complications.	4 weeks.	Not included in study.	No information about recurrence. Sample size was not calculated.
Xiong et al. 2009 [9]	RCT	56	I: 0.5 mL BCG-PSN injection every other day for 2 weeks. C: TA injections (0.5 mL, 20 mg/mL) with lidocaine every week for two weeks.	VAS; measuring the erosive area (mm^2^).	No statistical differences between two groups in erosive areas and VAS scores.	Swelling or burning sensation in 9.7% od BCG group and 8.0% in TA group.	3 months.	BCG-PSN 33% vs. 45.5% in TA; no statistical difference in intervals.	No objective scoring system to assess healing. Sample size was not calculated.
Metwalli et al. 2018 [10]	RCT	26	I: 0.5 mL BCG-PSN injection every other day for 2 weeks. C: injection of TA (20 mg/mL) with lidocaine; multiple 0.2 mL injections once weekly for 2 weeks.	Measuring the erosive area; REU scoring system; NRS.	No statistically significant differences between two groups in the reduction in REU scores and numerical rating score.	15.4% in TA group (atrophy and persistent erythema) vs. 23.1% in BCG-PSN group (swelling at the injection sites).	3 months	Nonsignificant difference.	Sample size was not calculated.
Lee et al. 2013 [11]	RCT	40	I: intralesional injection of TA (0.5 mL, 40 mg/mL), once a week for 4 weeks, and 1 injection after another 2 weeks. C: 0.4% mouthrinse of TA, 3 times daily for 3 weeks.	VAS; OHIP-14; scoring system of OLP by Escudier.	Similar, significant reduction in pain in both groups.	mouthrinse—44% candidiasis. injections—5% Cushingoid features.	1 year.	27.8% for mouthrinse; 40% for injections. Relapse time not significantly different.	Sample size was not calculated.
Liu et al. 2013 [12]	RCT	61	I: 1.4 mg intralesional betamethasone once a week for two weeks. C: 8 mg intralesional TA once a week for two weeks.	Measuring the erosive area (mm^2^); NRS.	Betamethasone was superior in reducing ulcer size and healing. No significant difference in pain reduction.	Betamethasone—burning sensation in the throat 6 h after injection (one patient).	3 months.	Relapse rate significantly lower in betamethasone group (14.8% vs. 45% in TA group).	Sample size was not calculated. Short follow-up.
Ahuja et al. 2020 [13]	RCT	20	I: intralesional PRP (0.5 mL/1 cm^2^ of lesion). C: intralesional TA (10 mg/mL) bilaterally; 0.5 mL for every 1 cm^2^ of lesion. Weekly injections for 2 months in both groups.	VAS; score 0–3 for erythema and lesion size.	Similar, significant reduction in pain, size od lesions, and erythema scores.	Mild side effects in 20% of TA group.	4 months.	Lower recurrence in PRP group (10% vs. 30%).	Sample size was not calculated. Non-objective scoring system.
ElGhareeb et al. 2022 [14]	RCT	24	I: injection of PRP. C: injection of TA (20 mg/mL) with lidocaine; multiple 0.2 mL injections. Injection every two weeks for two months in both groups.	REU; NRS; measuring lesion area (mm^2^).	No significant differences in REU and NRS in both groups; significant decrease in both groups.	More side effects in PRP group (especially pain).	3 months.	Higher recurrence in PRP patients (100% in 6 weeks).	Sample size was not calculated. Pain in PRP group may be due to lack of anesthesia.
Bennardo et al. 2021 [15]	RCT	9	I: 1 mL PRF injection in one buccal side. C: 0.5 mL TA (40 mg/mL) injection in opposite side.Once a week for a month on both sides.	VAS; Thongprasom score for morphological aspects of lesions; measuring the lesion area using Adobe Photoshop.	No statistically important differences between groups; both methods were effective.	Not mentioned.	8 weeks.	Not mentioned.	Sample size was not calculated. Split-mouth study is limited for pain evaluation.
Al-Hallak et al. 2022 [16]	RCT	12	I: 1 mL PRF injection in one buccal side. C: 0.5 mL TA (40 mg/mL) injection in opposite side. Once a week for a month.	VAS; REU score.	No statistically important differences between groups; both methods were effective, but TA showed more effectiveness.	Not mentioned.	3 months.	16.7% on both sides.	Sample size was not calculated. No information about side effects.
Agha-Hosseini et al. 2021 [17]	RCT	28	I: one buccal side—TA (40 mg/mL) with HA 7 mg. C: other buccal side—TA alone; 1 mL for every 2 cm^2^ area of the lesion in both groups.	VAS; Thongprasom’s scale.	No difference in pain level. Better resolution of lesions and symptoms for TA+HA.	Not mentioned.	6 months.	74% TA; 11.1% TA+HA.	No information about adverse effects.
Zhao et al. 2022 [18]	RCT	48	I: TA injection (0.5 mL, 20 mg/mL), periodontal scaling, and oral hygiene instruction. C:TA injection alone. Injection once a week for 2 weeks in both groups.	Measuring erosion size (mm^2^); NRS; PI; CPI.	Higher healing rate, and greater reduction in erosion size and pain level in experimental group.	1 patient in experimental group—mild dry mouth 4 h after injection.	3 months.	No difference in both groups.	Reduction in atrophic or white striae not included.
Kuo et al. 2013 [19]	Observational interventional study	50	TA injections (2 mL, 20 mg/mL once weekly for 2–3 weeks) plus oral administration of prednisolone (15–30 mg), then topical 0.1% dexamethasone and vitamin B9.	Division of lesions into categories using clinical examination.	Complete response in 90% of patients and partial response in 10% of patients.	Not mentioned.	3–24 months.	All patients with complete response reported recurrence in 3–24 months.	Non-objective scoring system. Sample size was not calculated.
Lee et al. 2018 [20]	Prospective cohort	62	TA injections (40 mg/mL) once a week for 4–6 weeks.	VAS; OHIP; scoring system by Escudier.	Improvement in 80.6% of patients. TA injections were less effective in patients with lesions on the lips.	Not included.	1 year.	58%.	No control group included. Sample size was not calculated.
Walia et al. 2022 [21]	Prospective observational	52	Intralesional injection of TA (0.5 mL, 40 mg/mL) once a week for 4 weeks, followed by one injection in the 6th week along with TA mucosal paste (0.1%) and TAC ointment (0.03%) in tapering dose until the 8th week.	VAS; scoring scale for lesions by Thongprasom et al.	78.8% of patients showed complete remission of disease and 21% showed partial improvement. Significant improvement in VAS score and size of lesions.	Transient burning sensation and alternation in taste in a few patients. Candidiasis.	20 weeks.	41%.	No control group. Sample size was not calculated.
Kurt et al. 2019 [22]	Case series	3	Replacing amalgam restoration in two patients with 1–2 injections of TA (0.4 mL, 10 mg/mL).	Observation; information from the patients about symptoms.	Complete response in two patients; one patient lost to follow-up	Not mentioned.	1 month.	Neither of the two patients.	Very small sample. Non-objective scoring system.Short follow-up.

RCT: randomized clinical trial; I: intervention; C: control; TA: triamcinolone acetonide; VAS: visual analog scale; REU: reticular, erosive and ulcerative lesions scoring system; BCG-PSN: Bacillus Calmette-Guerin polysaccharide nucleic acid; NRS: numeric rating scale; OHIP-14: oral health impact profile-14; OLP: oral lichen planus; PRP: platelet-rich plasma; PRF: platelet-rich fibrin; HA: hyaluronic acid; PI: plaque index; CPI: community periodontal index; TAC: tacrolimus.

**Table 2:** Most commonly used topical corticosteroids in the treatment of oral lichen planus.

Corticosteroid	Potency (US Class)	Typical Concentration	Frequency/Duration
Clobetasol propionate	Super-potent (Class I)	0.025–0.05%	2–3 times/day, 3–5 min/application
Fluocinonide	High potency (Class II)	0.025–0.05%	5–10 times/day, 3–5 min/application
Triamcinolone acetonide	Medium-to-high potency (Class III) at 0.5% Medium (Class IV and V) at 0.025–0.1%	0.05–0.5%	3–10 times/day, 3–5 min/application

## Data Availability

No new data were created or analyzed in this study.

## Abbreviations

The following abbreviations are used in this manuscript:

BCG-PSN	Bacillus Calmette-Guerin polysaccharide nucleic acid
CPI	Community periodontal index
EOLP	Erosive oral lichen planus
HA	Hyaluronic acid
OLP	Oral lichen planus
PI	Plaque index
PRF	Platelet-rich fibrin
PRP	Platelet-rich plasma
TA	Triamcinolone acetonide
TAC	Tacrolimus
REU	Reticular, erosive, and ulcerative lesions scoring system
VAS	Visual analog scale
